# Decreased expression of prenyl diphosphate synthase subunit 2 correlates with reduced survival of patients with gastric cancer

**DOI:** 10.1186/s13046-014-0088-3

**Published:** 2014-10-22

**Authors:** Mitsuro Kanda, Shuji Nomoto, Hisaharu Oya, Ryoji Hashimoto, Hideki Takami, Dai Shimizu, Fuminori Sonohara, Daisuke Kobayashi, Chie Tanaka, Suguru Yamada, Tsutomu Fujii, Goro Nakayama, Hiroyuki Sugimoto, Masahiko Koike, Kenta Murotani, Michitaka Fujiwara, Yasuhiro Kodera

**Affiliations:** Department of Gastroenterological Surgery (Surgery II), Nagoya University Graduate School of Medicine, 65 Tsurumai-cho, Showa-ku, Nagoya, 466-8550 Japan; Center for Advanced Medicine and Clinical Research, Nagoya University Hospital, Nagoya, Japan

**Keywords:** Gastric cancer, Prenyl diphosphate synthase subunit 2, Expression, methylation, Subtype

## Abstract

**Background:**

Identification of novel molecular biomarkers will improve the management of patients with gastric cancer (GC). Prenyl diphosphate synthase subunit 2 (PDSS2) is required for coenzyme Q10 biosynthesis and acts as a tumor suppressor; however, the role and regulatory mechanisms of *PDSS2* in GC are not understood. The aim of this study was to determine expression status and regulatory mechanisms of *PDSS2* in GC.

**Methods:**

Associations between expression and methylation of *PDSS2* were evaluated using GC cell lines. The clinical significance of *PDSS2* expression was evaluated using 238 pairs of surgically resected gastric tissues with subgroup analysis based on GC subtypes.

**Results:**

The expression of *PDSS2* mRNA was decreased in 73% of GC cell lines compared with the control non-cancerous cell. The *PDSS2* promoter was hypermethylated in cells with decreased *PDSS2* expression, and treating these cells with a methylation inhibitor reactivated *PDSS2* expression. GC tissues expressed significantly lower mean levels of *PDSS2* mRNA compared with adjacent normal tissues (*P* <0.001). The expression pattern of PDSS2 protein was consistent with that of its mRNA. The decrease of *PDSS2* mRNA expression in GC tissues (less than half the level of expression detected in the corresponding normal adjacent tissues) correlated significantly with elevated levels of carbohydrate antigen 19-9 (*P* = 0.015), lymph node metastasis (*P* = 0.022), and shorter recurrence-free survival after curative resection (*P* = 0.022). Further, multivariate analysis identified *PDSS2* mRNA expression as an independent prognostic factor (hazard ratio 1.95, 95% confidence interval 1.22–3.09, *P* = 0.005), and its expression pattern and prognostic significance were similar among three GC subtypes.

**Conclusions:**

*PDSS2* encodes a putative tumor suppressor, and we show here that its expression was regulated by hypermethylation of its promoter in GC cells. Inhibition of *PDSS2* mRNA expression may serve as a novel biomarker of all types of GC.

**Electronic supplementary material:**

The online version of this article (doi:10.1186/s13046-014-0088-3) contains supplementary material, which is available to authorized users.

## Background

Although the incidence of gastric cancer (GC) is declining in most developed countries, it remains one of the most common causes of cancer-related death worldwide [1-3]. Appropriate stratification of patients is a pivotal aspect of individualized treatment, leading to reducing mortality from this cancer [4,5].

According to its epidemiology, pathology, and location in the body, GC is recognized as three distinct malignancies arising in the same organ [6-8]. Shah et al. [9] proposed a convincing classification of GC according to histopathologic and anatomic criteria as follows: (1) proximal nondiffuse GC where the tumor is located mainly in the gastric cardia with evidence of precursor glandular dysplasia or in situ carcinoma in the presence of chronic inflammation, usually without atrophy; (2) diffuse GC, which may be located anywhere in the stomach with no apparent gastritis that exhibits an entirely diffuse pattern of infiltration of cells with a poorly differentiated phenotype; and (3) distal nondiffuse GC, which is located mainly in the distal stomach with evidence of chronic gastritis that is predominantly differentiated or exhibits an intestinal phenotype. In this study, they demonstrated that the three GC subtypes are distinguished by their gene expression profiles. Therefore, the genetic diversity of GC subtypes should be considered in studies of genetic and epigenetic alterations related to gastric carcinogenesis and progression.

Prenyl diphosphate synthase subunit 2 (*PDSS2*) was identified in 2005 [10], and evidence indicates that it acts as a tumor suppressor [11,12]. *PDSS2* is required for the synthesis of coenzyme Q10 (CoQ10) [13,14], which is synthesized in the mitochondrial inner membrane and plays a vital role in the mitochondrial respiratory chain, pyrimidine nucleoside biosynthesis, and modulation of apoptosis [15]. *PDSS2* resides within the chromosomal locus 6q16.3-21, a site of frequent microsatellite DNA instability and loss of heterozygosity (LOH) in GC [16,17], supporting its role as a tumor suppressor in gastric epithelial cells. Moreover, *PDSS2* may suppress the development of malignant melanomas and lung cancers [11,12]. Moreover, Chen et al. reported that enforced overexpression of *PDSS2* leads to apoptosis in a GC cell line by causing cell cycle arrest in the G0/G1 phase [18]. These reports led us to make a hypothesis that *PDSS2* is a potential GC-related gene and a candidate of novel clinically-relevant prognostic marker of GC.

In this study, expression and methylation status of *PDSS2* in GC were determined to evaluate the clinical significance and regulatory mechanisms of *PDSS2* expression in GC. Our results indicate that *PDSS2* expression provides a potential clinical biomarker of the progression and recurrence of GC.

## Material and methods

### Ethics

This study conformed to the ethical guidelines of the World Medical Association Declaration of Helsinki‐Ethical Principles for Medical Research Involving Human Subjects and has been approved by the Institutional Review Board of Nagoya University, Japan. Written informed consent for usage of clinical samples and data, as required by the institutional review board, was obtained from all patients [4].

### Sample collection

Eleven GC cell lines (H111, KATOIII, MKN1, MKN28, MKN45, MKN74, NUGC2, NUGC3, NUGC4, SC-2-NU and SC-6-LCK) and CCL-241 (non-cancerous cell line derived from the small intestine) were obtained from the American Type Culture Collection (Manassas, VA, USA) or Tohoku University, Japan. The GC cell lines were cultured at 37°C in RPMI-1640 (Sigma-Aldrich, St. Louis, MO, USA) supplemented with 10% fetal bovine serum in an atmosphere containing 5% CO_2_. For CCL-241, 30 ng/ml of the epidermal growth factor (Sigma-Aldrich) was added in the medium. Primary GC tissues and corresponding normal adjacent tissues were collected from 238 patients who underwent gastric resection for GC at Nagoya University Hospital between 2001 and 2012. Patients who received neoadjuvant therapy were excluded because it was difficult to obtain cancer cells from scarred tissues. Specimens were classified histologically using the 7th edition of the Union for International Cancer Control (UICC) classification [19]. Relevant clinicopathological parameters were acquired from medical records. To evaluate whether the expression level of *PDSS2* correlated with tumor phenotype, patients were categorized into three groups according to the definition of GC subtypes according to the criteria of Shah et al. [9] as follows: proximal nondiffuse, diffuse, and distal nondiffuse type. Since 2006, adjuvant chemotherapy using S-1 (an oral fluorinated pyrimidine) is administered to all UICC stage II–III patients with GC unless contraindicated by the patient’s condition [20]. Patients were followed at least once every 3 months for 2 years after surgery and then every 6 months for 5 years or until death. Physical examination, laboratory tests, and enhanced computed tomography (chest and abdominal cavity) were performed at each visit. Chemotherapy for patients with distant metastasis or after recurrence was determined by physician’s discretion.

Tissue samples were immediately frozen in liquid nitrogen and stored at −80°C. Tumor samples without necrotic areas (approximately 5 mm^2^) were extracted by gross observation and only samples confirmed to comprise more than 80% tumor components by H&E staining were included in this study. Corresponding normal adjacent gastric mucosa samples >5 cm from the edge of the tumors were obtained from the same patient [21].

### Quantitative real-time reverse-transcription polymerase chain reaction (qRT-PCR)

Total RNAs (10 μg per sample) were isolated from 11 GC cell lines, CCL-241, 238 primary GC tissues and corresponding normal adjacent tissues were used to generate cDNAs, which were amplified using specific PCR primers (Additional file 1: Table S1). Real-time detection of SYBR® Green fluorescence intensity was conducted using an ABI StepOnePlus Real-Time PCR System (Applied Biosystems, Foster City, CA, USA). The expression of *GAPDH* mRNA was quantified in each sample for standardization. The qRT-PCR reactions in each sample were performed in triplicate. The expression level of each sample is presented as the value of the *PDSS2* amplicon divided by that of *GAPDH* [22]. *PDSS2* mRNA expression was defined as decreased in GC tissues when its level was less than half that of the corresponding normal adjacent tissue.

### Analysis of the promoter region of *PDSS2*

The nucleotide sequence of the *PDSS2* promoter region was analyzed to determine the presence or absence of CpG islands defined as follows: at least a 200-bp region of DNA with a high GC content (>50%) and an Observed CpG/Expected CpG ratio ≥0.6 [23]. We used CpG Island Searcher software (http://cpgislands.usc.edu/) to determine the locations of CpG islands [24].

### Methylation-specific PCR (MSP) and bisulfite sequence analysis

*PDSS2* possesses a CpG island near its promoter region, and we hypothesized that aberrant methylation is responsible for regulating the transcription of *PDSS2* in GC. DNA samples from 11 GC cell lines treated with bisulfite were subjected to MSP and nucleotide sequence analysis [25]. The primer sequences used for MSP and bisulfite sequencing are listed in Additional file 1: Table S1.

### 5-Aza-2′-deoxycytidine (5-aza-dC) treatment

To assess the relation of promoter hypermethylation to *PDSS2* transcription, GC cells (1.5 × 10^6^) were treated with 5-aza-dC (Sigma-Aldrich) to inhibit DNA methylation and cultured for 6 days with medium changes on days 1, 3, and 5. RNA was extracted, and RT-PCR was performed as described [7].

### Immunohistochemistry (IHC)

IHC analysis of the localization of PDSS2 was performed using a mouse monoclonal antibody against PDSS2 (ab119768, Abcam, Cambridge, UK) diluted 1:150 in antibody diluent (Dako, Glostrup, Denmark) to probe 30 representative formalin-fixed and paraffin-embedded sections of well-preserved GC tissue described previously [3]. Staining patterns were compared between GCs and the corresponding normal adjacent tissues. To avoid subjectivity, the specimens were randomized and coded before analysis by two independent observers who were unaware of the status of the samples. Each observer evaluated all specimens at least twice to minimize intra-observer variation [7].

### Evaluation of the clinical significance of *PDSS2* expression

Correlations between the pattern of *PDSS2* mRNA expression and clinicopathological parameters were evaluated according to the differences among the three GC subtypes. Subgroup analysis of survival according to GC subtype was performed to determine the influence of *PDSS2* expression on patients’ outcomes.

### Statistical analysis

Relative levels of mRNA expression (*PDSS2/GAPDH*) between GC and adjacent normal tissues were analyzed using the Mann–Whitney *U* test. The *χ*^2^ test was used to analyze the significance of the association between the expression and methylation status of *PDSS2* and clinicopathological parameters. Disease-specific and disease-free survival rates were calculated using the Kaplan–Meier method, and the difference in survival curves was analyzed using the log-rank test. We performed multivariate regression analysis to detect prognostic factors using the Cox proportional hazards model, and variables with *P* < 0.05 were entered into the final model. All statistical analyses were performed using JMP 10 software (SAS Institute Inc, Cary, NC, USA). A value of *P* < 0.05 was considered statistically significant.

## Results

### Identification of a CpG island in the *PDSS2* promoter

**Figure 1:**
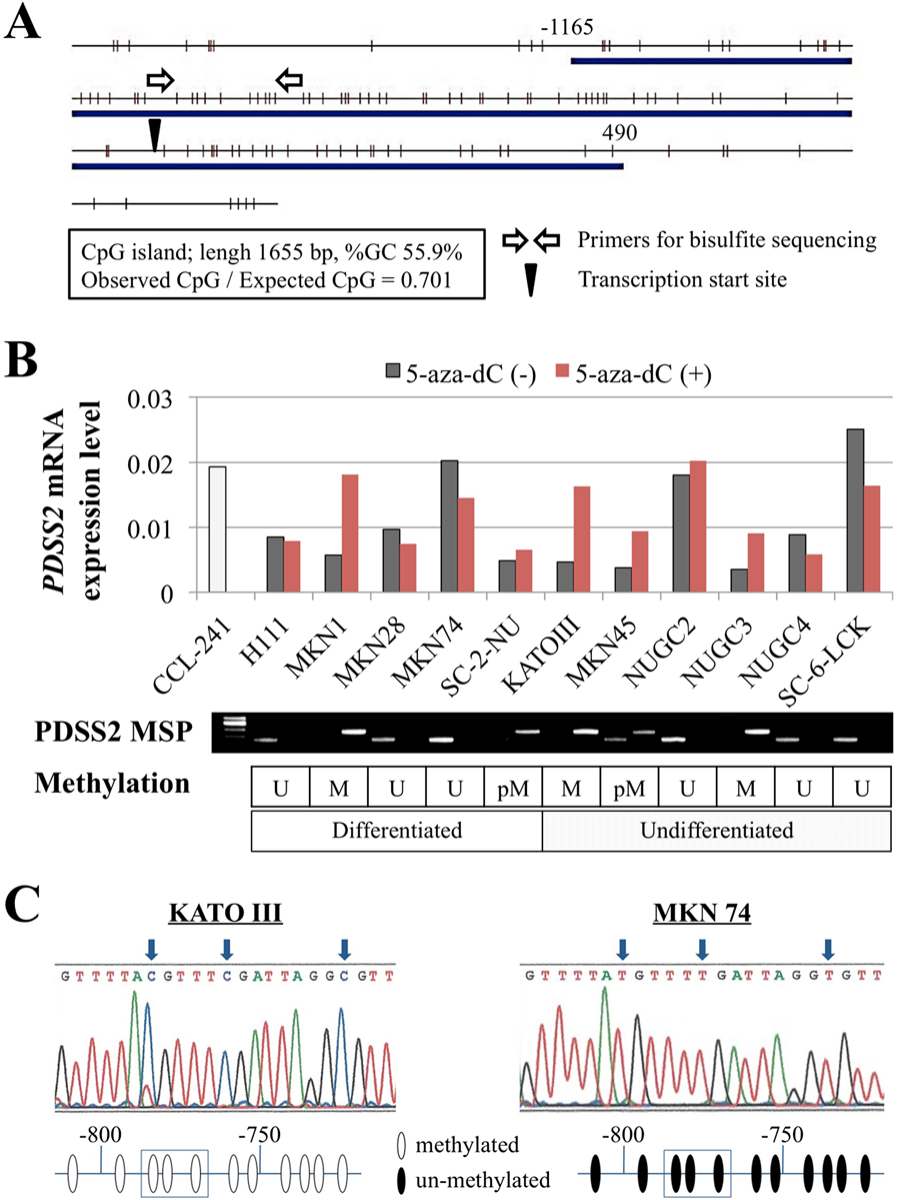
**Methylation analyses of PDSS2 in GC cell lines. (A)** The CpG island indicated by the blue line is centered on the *PDSS2* transcription initiation site extending upstream into the promoter region. **(B)** Bar graphs indicate *PDSS2* mRNA expression levels in CCL-241 (control non-cancerous cell) and GC cell lines before or after 5-aza-dC treatment. The methylation status of the *PDSS2* promoter was evaluated using MSP, and the results are enclosed in the box. M, methylated; pM, partially methylated; U, unmethylated. **(C)** Representative results of bisulfite sequence analysis. All CpG sites in KATOIII cell were retained as CG and those of MKN74 were converted to TG.

A CpG island was identified in the *PDSS2* promoter region using the CpG Island Searcher. The properties of the CpG island are as follows: 1655 bp, 55.9% GC, and 0.70 Observed CpG/Expected CpG ratio (Figure 1A). Therefore, we hypothesized that hypermethylation of the CpG islands regulates the expression of *PDSS2* in GC.

### *PDSS2* mRNA expression and methylation status in GC cell lines

Significant decreases in *PDSS2* mRNA levels were detected in seven (73%) of 11 GC cell lines compared with the expression level of the CCL-241 cell (Data for GC tissues are described below). There was no apparent difference in expression levels between cell lines derived from differentiated and undifferentiated GCs (Figure 1B). Hypermethylation of the *PDSS2* promoter was detected in MKN1, SC-2-NU, KATOIII, MKN45, and NUGC3 cells (Figure 1B). To determine whether hypermethylation of the *PDSS2* promoter inhibited transcription, mRNA expression levels were compared before and after treating cells with the methylation inhibitor 5-aza-dC. *PDSS2* mRNA levels were restored in cells with down-regulated *PDSS2* expression accompanying hypermethylation after 5-aza-dC treatment (Figure 1B), indicating that promoter hypermethylation inhibited *PDSS2* transcription in GC. Representative chromatograms of the sequence analysis of the *PDSS2* promoter region in MKN28 (complete methylation) and NUGC4 (absence of methylation) cells are shown in Figure 1C.

### Patient characteristics

The patient population included 179 males and 59 females aged from 20 to 84 years (65.3 ± 11.7 years, mean ± standard deviation). Pathologically, 139 and 99 patients were diagnosed with undifferentiated and differentiated GC, respectively. Patients were classified into the three GC phenotypes as follows: nondiffuse, 54; diffuse, 48; and distal nondiffuse, 136. According to the 7th edition of the UICC classification, 58, 40, 71 and 69 patients were in stages I, II, III and IV, respectively. One hundred sixty-four patients in stages I–III underwent R0 resection. Sixty of the 69 patients in UICC stage IV were diagnosed as stage IV due to positive peritoneal lavage cytology, localized peritoneal metastasis or distant lymph node metastasis. Eight of patients in stage IV had synchronous liver metastasis, one had lung metastasis, and they underwent gastrectomy aimed to control bleeding or obstruction to the passage of food.

### Expression levels of *PDSS2* mRNA and protein in surgically resected tissues

**Figure 2:**
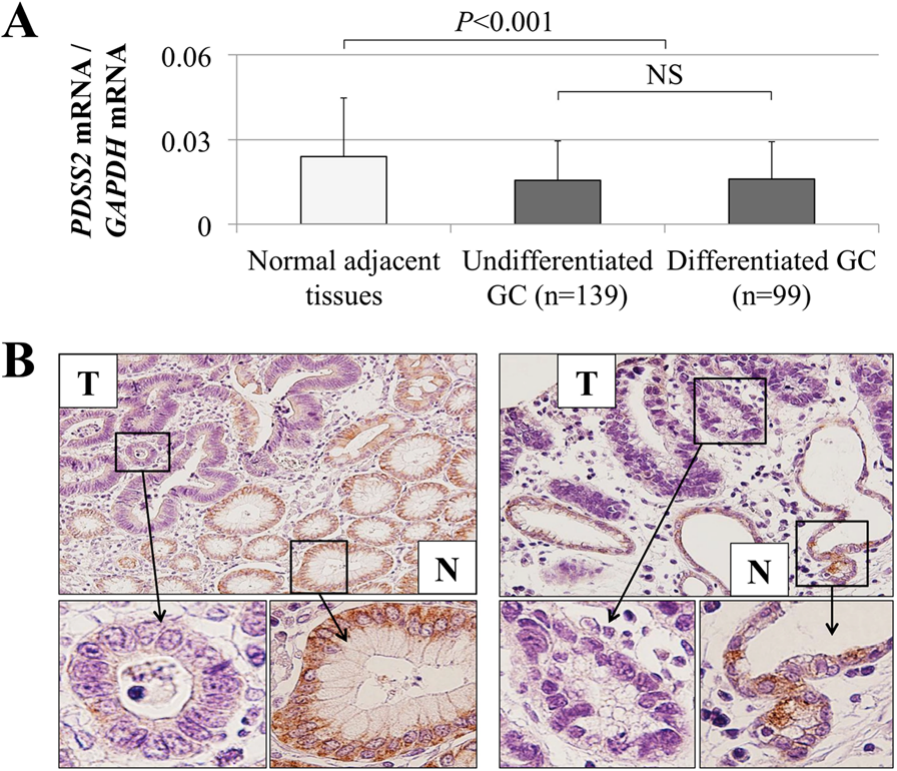
**Expression analyses of** ***PDSS2*** **in clinical specimens. (A)** The mean level of *PDSS2* mRNA in GC tissues compared with the corresponding normal adjacent tissues and in GC tissues between patients with undifferentiated GC and differentiated GC. **(B)** Representative IHC data comparing PDSS2 expression in tumor and adjacent normal adjacent tissue (magnified 100× and 400×). N, normal adjacent tissue; T, tumor tissue.

The mean expression level of *PDSS2* mRNA was lower in GC tissues compared with that of normal adjacent tissues (*P* <0.001); however, there was no significant difference in *PDSS2* mRNA expression levels between patients with undifferentiated or differentiated GC (Figure 2A). The expression pattern of PDSS2 was evaluated using IHC. Representative cases with reduced PDSS2 staining of GC tissues are shown in Figure 2B. Overall, the staining patterns of PDSS2 were consistent with the qRT-PCR data.

### Prognostic implications of *PDSS2* mRNA expression levels

**Figure 3:**
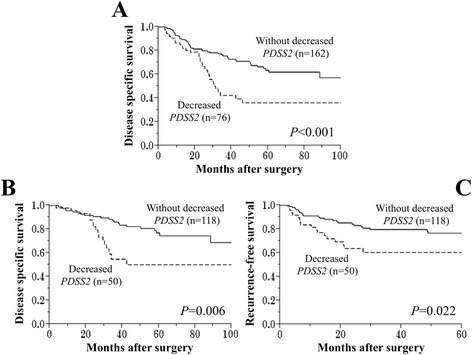
**Prognostic implications of** ***PDSS2*** **mRNA expression in patients with GC. (A)** Disease-specific survival of patients with decreased *PDSS2* mRNA in GC tissue. **(B) (C)** Disease-specific **(B)** and recurrence-free **(C)** survival among 168 patients who underwent R0 resection.

The expression of *PDSS2* mRNA in GC tissues was decreased in 76 (32%) of 238 patients (less than half the level of expression detected in the corresponding normal adjacent tissues). The disease-specific survival rate of patients with decreased levels of *PDSS2* mRNA in GCs was significantly lower compared with those without (5-year survival rates, 36% and 64%, respectively, *P* <0.001, Figure 3A). Decreased levels of *PDSS2* mRNA in GCs were significantly associated with carbohydrate antigen (CA) 19-9 > 37 IU/ml and lymph node metastasis (Table 1). Univariate analysis of disease-specific survival showed that GC subtype (proximal nondiffuse or diffuse), CA 19-9 > 37 IU/ml, tumor size (≥50 mm), pT4, undifferentiated tumor, lymphatic involvement, vessel invasion, invasive growth, lymph node metastasis, positive peritoneal lavage cytology, and decreased *PDSS2* mRNA expression in GC tissues were significant prognostic factors of adverse outcomes. Multivariate analysis identified decreased *PDSS2* mRNA expression as an independent prognostic factor (hazard ratio 1.95, 95% confidence interval 1.22–3.09, *P* = 0.005, Table 2). The proportional hazards assumption in the Cox model was assessed with models including time-by-covariate interactions and no significant violations were found in the model.

**Table 1:** **Association between expression level of** ***PDSS2*** **mRNA and clinicopathological parameters of 238 patients**

**Variables**	**Decreased** ***PDSS2*** **mRNA in GC tissues (n)**	**Others (n)**	***P*** **value**
Age			0.408
< 65 year	29	71	
≥ 65 year	47	91	
Gender			0.551
Male	59	120	
Female	17	42	
Subtype			0.298
Proximal nondiffuse	22	32	
Diffuse	14	33	
Distal nondiffuse	40	96	
Carcinoembryonic antigen (ng/ml)			0.490
≤ 5	59	132	
> 5	17	30	
Carbohydrate antigen 19-9 (IU/ml)			0.015*
≤ 37	55	139	
> 37	21	23	
Tumor size (mm)			0.104
< 50	29	80	
≥ 50	47	82	
Tumor depth (UICC)			0.419
pT1	14	32	
pT2	6	24	
pT3	19	33	
pT4	37	73	
Differentiation			0.217
Differentiated	36	63	
Undifferentiated	40	99	
Lymphatic involvement			0.201
Absent	8	27	
Present	68	135	
Vessel invasion			0.535
Absent	31	73	
Present	45	89	
Infiltrative growth type			0.443
Invasive growth	24	59	
Expansive growth	52	102	
Lymph node metastasis (UICC)			0.022*
pN0	19	70	
pN1	8	19	
pN2	12	24	
pN3	37	49	
Peritoneal lavage cytology			0.306
Negative	57	131	
Positive	19	31	
Distant metastasis (UICC)			0.228
M0	50	119	
M1	26	43	

*Statistically significant (*P* <0.05). GC, gastric cancer; UICC, Union for International Cancer Control.

**Table 2:** **Prognostic factors for disease-specific survival of 238 patients**

**Variables**	**n (%)**	**Univariate**			**Multivariable**		
		**Hazard ratio**	**95% CI**	***P*** **value**	**Hazard ratio**	**95% CI**	***P*** **value**
Age (≥65)	138 (58%)	1.04	0.67 – 1.61	0.843			
Gender (female)	59 (25%)	1.29	0.79 – 2.05	0.301			
Subtype (distal nondiffuse)	136 (57%)	0.43	0.28 – 0.67	<0.001	0.64	0.40 – 1.01	0.056
Carcinoembryonic antigen (>5 ng/ml)	47 (20%)	1.48	0.86 – 2.42	0.149			
Carbohydrate antigen 19-9 (>37 IU/ml)	44 (18%)	1.98	1.17 – 3.20	0.012	1.23	0.71 – 2.06	0.445
Tumor size (≥50 mm)	129 (54%)	2.86	1.78 – 4.80	<0.001	1.40	0.83 – 2.42	0.211
Tumor depth (pT4, UICC)	110 (46%)	4.17	2.61 – 6.88	<0.001	1.38	0.78 – 2.50	0.273
Tumor differentiation (undifferentiated)	139 (58%)	2.03	1.28 – 3.32	0.002	1.45	0.83 – 2.60	0.197
Lymphatic involvement	203 (85%)	6.31	2.36 – 25.8	<0.001	1.45	0.45 – 6.50	0.559
Vessel invasion	134 (56%)	2.65	1.66 – 4.37	<0.001	1.75	1.07 – 2.97	0.026*
Invasive growth	83 (35%)	3.03	1.97 – 4.70	<0.001	1.19	0.70 – 2.05	0.520
Lymph node metastasis	149 (63%)	7.02	3.70 – 15.1	<0.001	1.38	0.56 – 3.81	0.503
Peritoneal lavage cytology (positive)	50 (21%)	4.33	2.76 – 6.74	<0.001	1.87	1.14 – 3.06	0.014*
UICC stage (III-IV)	140 (59%)	9.68	4.97 – 21.8	<0.001	2.63	0.94 – 7.83	0.065
Decreased *PDSS2* mRNA in GCs	76 (32%)	2.18	1.40 – 3.37	<0.001	1.91	1.19 – 3.04	0.008*

*Statistically significant in multivariate analysis. GC, gastric cancer; CI, confidence interval; UICC, Union for International Cancer Control.

Of the 168 patients who underwent R0 resection, the disease-specific survival rate was significantly lower for those with decreased *PDSS2* mRNA expression in GCs (n = 50) compared with those without (n = 118) (5-year survival rates, 50% and 77%, respectively, *P* = 0.006, Figure 3B). Patients with decreased *PDSS2* mRNA expression in GCs experienced significantly earlier recurrences after surgery compared with those without (2-year recurrence-free survival rates, 64% and 84%, respectively, *P* = 0.022, Figure 3C). Initial recurrence sites of 43 relapsing patients with decreased *PDSS2* mRNA expression in GCs were peritoneal in 21 (49%), liver in 6 (14%), lymph node in 13 (30%) and others (e.g. lung and bone) in 3 patients. On the other hand, those of 61 relapsing patients without decreased *PDSS2* were peritoneal in 35 (57%), liver in 10 (16%), lymph node in 8 (13%) and others in 8 patients. Patients with decreased *PDSS2* mRNA expression in GCs were likely to have a node relapse, though it did not reach the statistical significance.

### Subgroup analysis of *PDSS2* expression according to GC subtype

**Figure 4:**
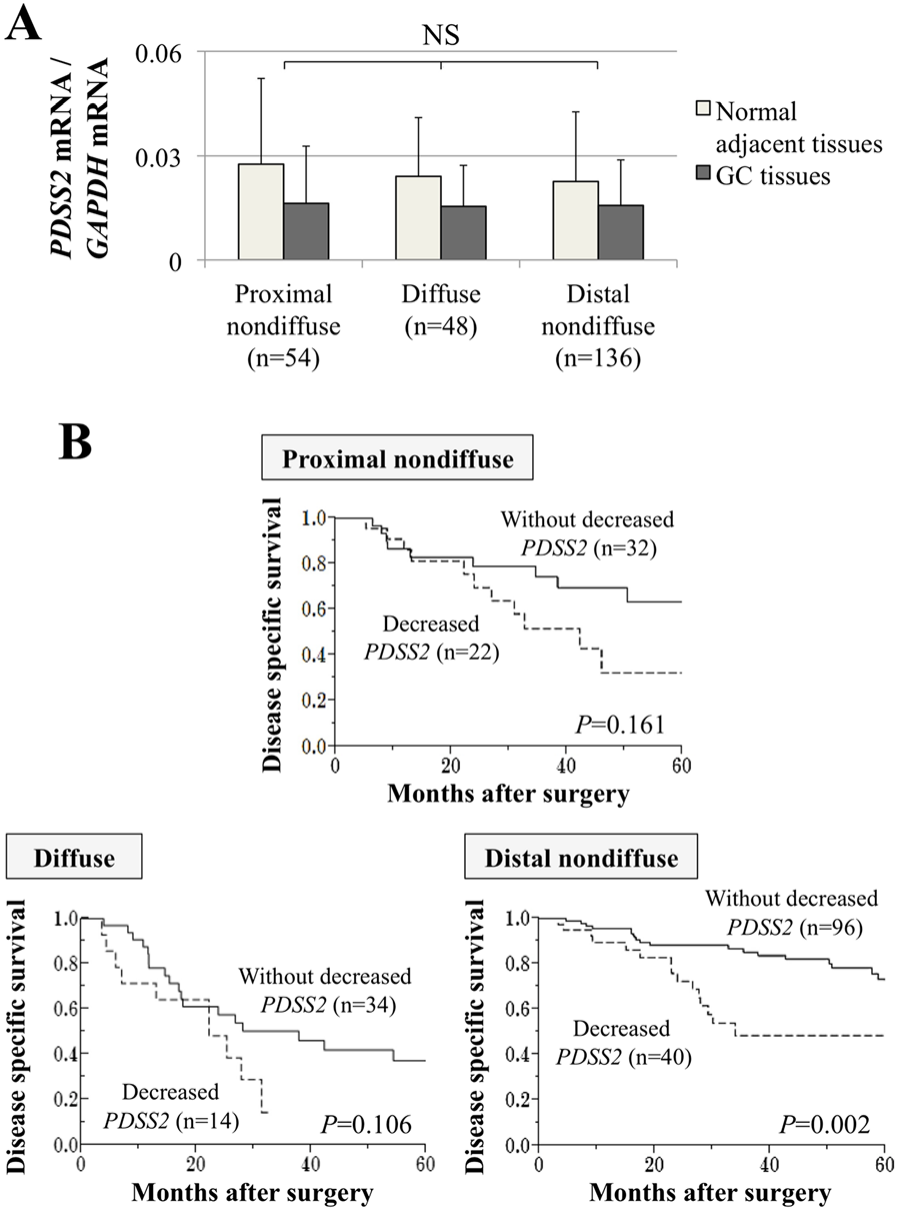
**Subgroup analysis of GC subtypes. (A)** *PDSS2* mRNA expression levels among the three GC subtypes both in GC and normal adjacent tissues. **(B)** Comparison of disease-specific survival of patients with and without decreased *PDSS2* mRNA expression in GCs of each GC subtype.

Mean *PDSS2* mRNA expression levels were equivalent in GC and normal adjacent tissues (Figure 4A). Similarly, the prognostic value of decreased *PDSS2* mRNA expression in GCs was comparable among the three GC subtypes (Figure 4B).

## Discussion

PDSSs are heterotetrameric enzymes comprising subunits encoded by *PDSS1* (10p12.1) and *PDSS2* [10,12]. PDSS activity requires both subunits [10,14,15]. The association of *PDSS2* with GC was considered because of its chromosomal location (6q21), and because of its inactivation or loss from certain malignancies [16,26]. Here we show that *PDSS2* mRNA was heterogeneously expressed in GC cell lines, and its expression was inhibited in 73% and 32% of GC cell lines and tumor tissues, respectively. We detected hypermethylation of the *PDSS2* promoter in five (45%) of 11 GC cell lines with significantly decreased levels of *PDSS2* expression. Further, *PDSS2* transcription was reactivated after cells were treated with an inhibitor of DNA methylation. These findings are the first to our knowledge to show that promoter hypermethylation regulates *PDSS2* transcription. However, *PDSS2* expression was decreased in some GC cells without hypermethylation. Because chromosome 6q is a frequent site of LOH in GC [17,26,27], LOH may regulate *PDSS2* expression as well.

There has been a report demonstrating that *PDSS2* was expressed at decreased or undetectable expression in a small number of biopsy GC samples [18]. In the present study, we analyzed 238 surgical specimens of tumors and the corresponding uninvolved tissue to gain further insight into the clinical significance of *PDSS2* expression in GC. Consistent with analyses of malignant melanoma and lung cancer [11,12], most patients with GC harbored a decreased level of *PDSS2* mRNA in GC tissues, and the mean *PDSS2* expression level was significantly decreased in GC compared with normal adjacent tissues. IHC was conducted to determine whether the mRNA level reflected *PDSS2* protein expression. Because the IHC results indicated that the mRNA data were consistent with the protein level, subsequent analyses were performed according to the mRNA data, which are more amenable to the quantitative analysis [7,23].

Decreased *PDSS2* mRNA expression in GCs was significantly associated with elevated preoperative CA19-9 levels and lymph node metastasis and was identified as an independent prognostic factor. Moreover, patients with decreased *PDSS2* mRNA expression in GC tissue experienced significantly earlier recurrence after R0 resection. Recently, Chen et al. investigated the tumor-suppressing activity of *PDSS2* in lung cancer [28]. They reported that the forced overexpression of *PDSS2* caused massive cell death through apoptotic pathways and significantly inhibited colony formation and there was an inverse correlation between *PDSS2* expression and gelsolin expression, which is known to inhibit apoptosis and enhance cell invasion and metastasis [29], though PDSS2 did not influence the sensitivity of the cancer cells to chemotherapeutic drugs [28]. This tumor suppressive mechanism of *PDSS2* might be applied to GC as well.

The expression pattern of *PDSS2* mRNA and its prognostic impact were similar among the three GC subtypes (proximal nondiffuse, diffuse, and distal nondiffuse), indicating that *PDSS2* expression influences the pathogenesis of all types of GC. Shah et al. reported that one-third of amplified genes, possibly including *PDSS2*, showed equivalent expression pattern among the three GC subtypes [9].

GC is one of the tumors with a high frequency of aberrant methylation, and it frequently exhibits the CpG island methylator phenotype [30,31]. The expression of a large number of genes is suppressed by CpG island hypermethylation in GC cells, including those encoding tumor suppressors, cell cycle regulators, inducers and executioners of apoptosis, proteins that promote the invasive phenotype, and DNA mismatch repair enzymes [32]. These epigenetic alterations can serve as biomarkers that illuminate an increased metastatic potential and aggressive tumor phenotype [33] as well as therapeutic targets [34]. Therefore, identification of other genes that are regulated by methylation in GC cells will likely improve the management of GC.

The tumor suppressive function of *PDSS2* are supported by the present findings as follows: (1) decreased expression of *PDSS2* was frequently detected in GC tissues, (2) the mean level of *PDSS2* expression was significantly lower in GC tissues, and (3) decreased expression of *PDSS2* was associated with early recurrence and subsequent poor prognosis. *PDSS2* expression levels in biopsy tissue obtained using endoscopic surveillance samples or in surgical specimens may be useful for predicting early recurrence and poor prognosis, which will likely aid efforts to design more efficacious therapeutic strategies.

This study was limited by its lack of sufficient functional analysis of *PDSS2*, which tempers the conclusion that it acts as a tumor suppressor in GC. Further studies including pathway analysis in gastric carcinogenesis and functional analysis are expected to clarify the molecular mechanisms underlying the biological activities of *PDSS2* in GC.

## Conclusion

In conclusion, our findings support the conclusion that the expression of the putative tumor suppressor gene *PDSS2* is regulated by promoter hypermethylation in GC cells and indicate. Our results indicate further that decreased expression of *PDSS2* mRNA may represent a novel biomarker for progression and recurrence of all types of GC.

## Additional file

Additional file 1: Table S1. Primers and annealing temperature.
