# Supplementary material for: Decreased expression of prenyl diphosphate synthase subunit 2 correlates with reduced survival of patients with gastric cancer
Source: J Exp Clin Cancer Res. 2014 Oct 22;33(1):88. doi: 10.1186/s13046-014-0088-3 (PMC4209044; doi:10.1186/s13046-014-0088-3)
Supplement: Additional file 1: Table S1. — Primers and annealing temperature. [file 13046_2014_88_MOESM1_ESM.doc]

**TABLE S1 Primers and annealing temperatures**

| Gene | Experiment | Type | Sequence (5´ - 3´) | Product size | Annealing temperature |
| --- | --- | --- | --- | --- | --- |
| *PDSS2* | qRT-PCR | forward | GAATCAGGTAGTGTCAGAGG | 181 bp | 60 °C |
|  |  | reverse | GAGGCTATTCCAGCTGTCATG |  |  |
|  | MSP | forward | TCGAAGTTGGATTCGAGGAT | 263 bp | 62 °C |
|  | methylated | reverse | AAACGTCGAACGAAAACACC |  |  |
|  | MSP | forward | GGTTGGTGGTGATAGTGATA | 195 bp | 56 °C |
|  | un-methylated | reverse | CAACAAATAATCCCTCTACAC |  |  |
|  | Bisulfite | forward | TGTTTGGTTGGGTTTTGAGG | 152 bp | 58 °C |
|  | sequencing | reverse | CACCAACCCCTA ACA ATA AC |  |  |
| *GAPDH* | qRT-PCR | forward | GAAGGTGAAGGTCGGAGTC | 226 bp | 60 °C |
|  |  | probe | CAAGCTTCCCGTTCTCAGCC |  |  |
|  |  | reverse | GAAGATGGTGATGGGATTTC |  |  |

*PDSS2*, prenyl diphosphate synthase subunit 2; *GAPDH*, glyceraldehyde-3-phosphate dehydrogenase; qRT-PCR, quantitative real-time reverse-transcription polymerase chain reaction; MSP, methylation-specific PCR; bp, base pair.
